# Motivational dynamics of eating regulation: a self-determination theory perspective

**DOI:** 10.1186/1479-5868-9-21

**Published:** 2012-03-02

**Authors:** Joke Verstuyf, Heather Patrick, Maarten Vansteenkiste, Pedro J Teixeira

**Affiliations:** 1 Department of Developmental, Personality and Social Psychology, Ghent University, H. Dunantlaan 2, 9000 Ghent, Belgium; 2 National Cancer Institute, 6130 Executive Boulevard, Rockville, MD 20852-7335 USA; 3 Faculty of Human Kinetics, Technical University of Lisbon, Estrada da Costa, 1495-688 Cruz Quebrada, Portugal

**Keywords:** Eating Regulation, Eating Disorders, Self-Determination Theory, Motivation, Autonomous Regulation, Need Substitutes, Thin-Ideal

## Abstract

Within Western society, many people have difficulties adequately regulating their eating behaviors and weight. Although the literature on eating regulation is vast, little attention has been given to motivational dynamics involved in eating regulation. Grounded in Self-Determination Theory (SDT), the present contribution aims to provide a motivational perspective on eating regulation. The role of satisfaction and thwarting of the basic psychological needs for autonomy, competence, and relatedness is introduced as a mechanism to (a) explain the etiology of body image concerns and disordered eating and (b) understand the optimal regulation of ongoing eating behavior for healthy weight maintenance. An overview of empirical studies on these two research lines is provided. In a final section, the potential relevance and value of SDT in relation to prevailing theoretical models in the domain of eating regulation is discussed. Although research on SDT in the domain of eating regulation is still in its early stages and more research is clearly needed, this review suggests that the SDT represents a promising framework to more thoroughly study and understand the motivational processes involved in eating regulation and associated problems.

## Introduction

During the past half-century, the Western world has witnessed an intriguing paradox in the domain of eating regulation: an increase in body image concerns and restrictive eating [1,2] has occurred in conjunction with a dramatic rise in overweight and obesity [3]. Although somewhat ironic, this is not entirely surprising given the proliferation of conflicting advertisements for foods that are highly energy-dense and images of extraordinarily thin models in fashion and movies [4,5]. Failures in eating regulation have been found to culminate in a variety of physical and mental health risks. For instance, body image concerns are associated with more unhealthy weight control behaviors and lower well-being [6]. Problems in weight management, such as overweight and obesity, are associated with lower self-esteem and greater health risks (e.g., coronary heart disease) [7,8]. And disordered eating, such as more extreme forms of restrictive or disinhibited eating, and unhealthy weight control behaviors such as purging or use of laxatives or diuretics, are associated with a variety of psychological (e.g., low self-esteem) and health (e.g., heart failure) risks [9,10].

As a consequence of the high prevalence of problematic eating regulation and the psychological and physical health costs associated with these behaviors, several public health efforts have been launched to prevent and reduce these eating regulation problems. Further, in the academic arena, a number of theoretical models have been developed to study factors that contribute to the genesis and maintenance of these behaviors. Some models, like the Thin-Ideal Internalization Model [2] and Self-Objectification Theory [11] attempt to explain the etiology of body image concerns, while other models, such as the Dietary Restraint Theory [12] and the Self-Control Model [13], focus on the dynamics involved in failures of eating regulation for healthy weight management.

Eating regulation can encompass a range of behaviors and goals, such as choosing healthy foods, restrictive restraint, weight management, and disordered eating. Within each of these dimensions, some processes are likely to be more salient than others. For instance, a focus on the thin-ideal and perfectionist functioning might be more salient among anorectic women compared to binge eating women. Indeed, browsing through web of science, it becomes clear that more studies examined the role of the thin-ideal and perfectionism in anorectic eating behaviors compared to binge eating behaviors (61 versus 11 hits and 316 versus 63 hits). Similarly, autonomous motivated eating regulation is studied relatively frequent in groups of obese patients, whereas it has rarely been studied with eating disordered patients. In other words, research has remained somewhat divided across the diverse dimensions of eating behaviors, with specific theoretical perspectives being developed for specific eating behaviors [14]. Although it is important to examine which processes are more typical for specific eating behaviors, we maintain it is equally important to consider the more global motivational processes involved in eating regulation, an issue that can be addressed by relying on more general theories on motivation and personality development, like Self-Determination Theory (SDT) [15,16].

SDT offers a broader perspective on human functioning and has broad-reaching applications in a wide variety of contexts, such as education, exercise, work, relationships, psychopathology and psychotherapy [17,18]. SDT may also provide a framework to understand the myriad behaviors involved in eating regulation. Specifically, the concept of basic psychological needs, as conceived within SDT, can add to our understanding of the etiology of adaptive and disordered forms of eating regulation as well as to motivational processes involved in day-to-day eating regulation. Furthermore, although some motivational processes might be more salient in specific eating regulation problems, the consequences of the motivational basis for eating regulation would be similar across the range of eating behaviors. In general, need thwarting experiences relate to less adaptive and more disordered forms of eating regulation, whereas need satisfying experiences relate to more adaptive and less disordered forms of eating regulation. For instance, although a focus on the thin-ideal might be more salient among anorectic women, to the extent that obese women are focused on this ideal and need-thwarting experiences are provoked, we hypothesize similar maladaptive consequences of this motivational goal in this group of women. Therefore, the same motivational processes can relate to the understanding of models that have been developed for body image concerns (e.g., Thin-Ideal Internalization Model, Self-Objectification Theory) as well as to models developed for dieting, weight control, and binge eating behaviors (e.g., Dietary Restraint Theory, Self-Control Model).

**Figure 1:**
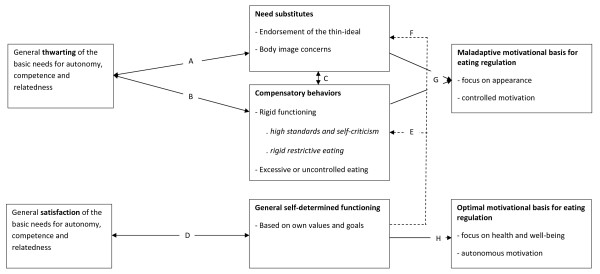
**The role of basic psychological need satisfaction versus thwarting in the etiology of disordered eating**.

**Figure 2:**
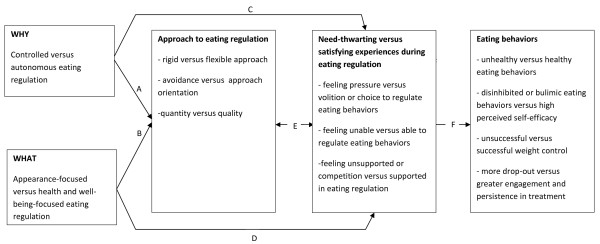
**The Role of Basic Psychological Need Satisfaction versus Thwarting in the Ongoing Regulation of Eating Behaviors**.

SDT is comprised of five different mini-theories [18], with some of them yielding more direct relevance for the understanding of eating regulation than others. Therefore, rather than presenting these five mini-theories in an exhaustive and theory-driven fashion, we chose to organize this paper around three larger sections, that is, (1) 'The Role of Psychological Needs in the Etiology of Disordered Eating '; (2) 'The Role of Psychological Needs in the Optimal Regulation of Eating Behaviors'; and (3) 'SDT in Relation to Current Perspectives on Body Image Concerns and Eating Regulation'. In the first part, we discuss the basic theoretical tenets of SDT and describe their relevance for the etiology of disordered eating (see Figure 1). Following the theoretical tenets, an overview of empirical evidence is provided and remaining research questions are addressed. In the second part, the relevance of SDT for the ongoing regulation of eating behaviors is discussed (see Figure 2), followed by a review of supporting empirical evidence and remaining research questions. Finally, in the third part, we briefly discuss the potential relevance and added value of SDT for some prevailing theoretical models in the domain of eating regulation.

### The Role of Psychological Needs in the Etiology of Disordered Eating

#### Need Substitutes and Compensatory Behaviors: A Self-Determination Theory Perspective

As a meta-theory of human motivation, SDT begins with three key assumptions. The first is that human beings are inherently proactive, that they have the potential to act on and master both the internal (i.e., drives and emotions) and the external (i.e., environmental) forces they encounter, rather than being passively controlled by those forces [16]. Second, SDT assumes that through their activity humans steadily move towards increasing levels of psychological growth and integration. Third, SDT acknowledges that, despite this innate tendency, characteristics of the social context may support or thwart growth and integration [16]. Thus, SDT integrates both the role of the person - their inner resources and capacity for growth - and the role of the social context in human motivation.

SDT has placed primary importance on psychological needs because the satisfaction or thwarting of these psychological needs plays a critical role in the process of growth and integration. Within the SDT framework, basic psychological needs are defined as the psychological nutriments necessary for growth and integration [16]. Using this definition, SDT has identified three basic needs: competence, relatedness, and autonomy. Competence reflects the need to feel efficacious and capable of achieving desired outcomes. Although not necessarily defined as an innate need, the issue of self-efficacy has been emphasized in many other theories of human motivation that have been applied to the study of eating regulation (e.g., social cognitive theory). Relatedness involves the need to feel close to and valued by important others, to have a sense of belonging with peers, family, and community. Finally, autonomy is the need to feel volitional, as the originator of one's actions in carrying out an activity. Just as the satisfaction of one's physiological needs (e.g., hunger) is critical for one's physical survival, the satisfaction of one's basic psychological needs is critical for psychological thriving and well-being [15].

Beyond theoretical conjecture, an impressive body of research conducted in various cultures with individuals across the life course has demonstrated the importance of need satisfaction for physical and mental health including higher well-being (e.g., life satisfaction, vitality), less ill-being (e.g., depression, anxiety), and better health [17,18]. Such findings have been reported at the interindividual level [19] and at the intrapersonal level, with diary studies demonstrating that daily well-being fluctuations co-vary with daily variation in the satisfaction of one's basic psychological needs [20].

The satisfaction versus thwarting of psychological needs is involved in the etiology of psychopathology [21]. When people chronically fail to have the three basic psychological needs met, they develop strategies to deal with this psychological deficit. Two maladaptive coping responses discussed within SDT involve the development of need substitutes and the engagement in compensatory behaviors [15,21] (see paths A and B in Figure 1).

##### Need Substitutes

Need substitutes are defined as goals that people engage in to compensate for a lack of experienced need satisfaction [15,22]. SDT distinguishes between extrinsic goals, such as popularity, physical attractiveness, and financial success, and intrinsic goals, such as personal growth, contributing to the community, personal relationships, and health. Extrinsic goals are very salient in a consumer culture, where fame, money, and the 'perfect body' [23,24] are portrayed as signs of success [25]. The appeal of such goals lies mainly in the anticipated power, social approval, or sense of worth that individuals expect from attaining them [26,27]. Although such goals or behaviors hold the promise of being satisfying and rewarding in the short term, they may interfere with genuine need satisfaction and therefore typically fail to yield long-term well-being benefits [28]. The experience of repeated need thwarting results in susceptibility to cultural messages touting that the pursuit and attainment of extrinsic goals brings happiness. Indeed, repeated need thwarting has been associated with feelings of insecurity and a resulting quest for external indicators of worth, which align with SDT's conceptualization of extrinsic goals [21]. Children raised in a social environment deprived of need support and nurturance are more likely to pursue extrinsic, relative to intrinsic, goals [29]. Also, children who feel unaccepted by their peers (i.e., thwarting of relatedness needs) experience more peer pressure to have the right 'stuff' and a stronger endorsement of materialistic values [30]. Importantly, not all extrinsic goals studied within SDT may be relevant in the context of eating regulation and body image concerns. Physical appearance and body image as need substitutes seem particularly relevant to eating regulation. For instance, people who adopt the thin-ideal, which represents a more extreme and socially prescribed form of physical attractiveness, experience more body image concerns and report more restrictive and problematic dietary behaviors [2].

##### Compensatory Behaviors

A second response to need thwarting involves the engagement in compensatory behaviors. Some people cope with need thwarting experiences by releasing or even revolting against self-control. For instance, need thwarting has been associated with alcohol abuse [31] and tobacco smoking [32]. In a similar vein, people may try to handle their need thwarting experiences by excessive eating or uncontrolled eating. The "escape-of-awareness" model [33] proposes that binge eating is a motivated attempt to escape awareness. To escape emotional distress, often provoked by high standards and self-criticism, binge eaters divert their attention away by narrowing the attention to immediate stimuli in the environment. Also in the affect regulation model [34], binge eating is considered a mechanism to cope with negative emotions. Similarly, the experience of need thwarting relates to excessive or uncontrolled eating because one tries to overcome negative affect associated with need thwarting experiences - in this case, using food as the substance of choice, analogous to what has been found with tobacco use and problem drinking.

Another compensatory behavior proposed within SDT is the development of rigid behavior patterns. People engage in such behaviors to obtain a sense of structure, predictability, and security in their lives. However, because people regulate their behavior in an inflexible and sometimes even compulsive fashion, they likely direct attention away from the deeper causes of their experienced need thwarting. In addition, they are prone to experience ill-being when they are unable to persist in their rigid functioning. An example of rigid behavioral patterns involves setting high, perfectionist standards. When confronted with the repeated failure to fulfill basic psychological needs, an individual might turn toward the pursuit of perfectionist standards in an attempt to prove one's worth to both oneself and one's surroundings. These high standards are pursued in a rigid fashion and are typically accompanied by dichotomous or "black-white" thinking [35]. Even a small failure to achieve these high standards gives rise to intense feelings of guilt, inferiority, and self-criticism. Instead, experiences of success are short-lived and are typically attributed to external and unstable causes (e.g., luck). Following success, individuals who hold perfectionist standards therefore typically raise their standards, thereby further reinforcing their relentless pursuit of perfection [35-37].

In the context of eating regulation, rigid behavior patterns may be characterized by extreme restriction with respect to portion sizes, calories and food types (e.g., eliminating or severely limiting a particular macro-nutrient such as fat or carbohydrates). Flexibility is not tolerated and even a small deviation from one's stringent eating routines gives rise to feelings of inferiority. Often when people subscribe to such restrictive eating practices, small deviations can quickly spiral into full-blown binges [38]. Consistent with this reasoning, both clinical accounts and empirical studies have provided strong and consistent evidence for an association between perfectionism and eating disorder pathology [39].

Need substitutes and compensatory behaviors often get intertwined in practice (path C in Figure 1). For instance, someone may rigidly stick to an extremely low-calorie diet (thereby engaging in a rigid behavioral pattern) with the aim of achieving the perfect body (thereby adopting a need substitute). In line with this idea, perfectionist strivings and the pursuit of the thin-ideal have been found to be interrelated [40]. Also uncontrolled eating has been associated with perfectionist standards and self-critical functioning [33] as well as with sticking to an extreme and rigid diet [12,41].

Although both compensatory behaviors and the attainment of need substitutes may engender some derivative satisfaction, such feelings fail to provide long-term benefits for wellbeing and growth as they are unlikely to satisfy psychological needs [28,42] (see reciprocal paths A and B in Figure 1). For instance, an anorectic person may derive a sense of competence from succeeding in extreme dietary restriction and weight loss goals. However, by adopting these rigid compensatory behaviors she diverts her attention away from deeper causes of need thwarting and her condition may block the experience of genuine competence satisfaction in other life domains. Further, the rigid focus on eating behaviors might provoke internal conflict and stress and likely disrupts relationships as well as the attainment of more intrinsic life goals, thereby engendering social isolation and autonomy thwarting. In other words, the pursuit of need substitutes and engagement in compensatory behaviors interferes with genuine need satisfaction [15,21], such that individuals get caught within an aggravating and negative cycle of need thwarting and eating pathology.

##### General Self-Determination

Until now we have described the effects of need thwarting on body image and disordered eating through the development of need substitutes and compensatory behavioral patterns. However, it is equally important to consider the benefits of need satisfaction - and not just the detriments of need thwarting. When basic needs are satisfied, people develop a more general self-determined orientation toward themselves and their social surroundings (see path D in Figure 1). General self-determination reflects the degree to which people function on the basis of their own interests, values and goals, whereas people who are less self-determined tend to be oriented more toward pressure and social expectations in their environment [18]. General self-determination can function as a buffer against sociocultural pressures to be thin and adopting the thin-ideal, which constitute risk factors for body image concerns and disordered eating [2,43] (see paths E and F in Figure 1). Further, people who are more self-determined are also more likely to engage in activities or goals that reflect their own interests and values, which in turn creates more opportunities for need-satisfying experiences (see reciprocal path D in Figure 1).

#### Need Substitutes and Compensatory Behaviors: An Overview of Empirical Evidence

A growing number of studies support the role of need thwarting in the etiology of endorsing the thin ideal, body image concerns, and subsequent eating disorder-related symptoms. A first group of studies focused on the support and thwarting of the psychological needs within the family context as an antecedent to need satisfaction, rigid behavior patterns and disordered eating (see paths A and B in Figure 1). For instance, Thøgerson-Ntoumani et al. [44] found that parental need support was associated with greater experienced need satisfaction which, in turn, was predictive of fewer body image concerns and unhealthy weight behaviors, such as skipping meals and purging. Soenens and colleagues [45] studied the associations between psychologically controlling parenting, perfectionism, and eating disorder outcomes in a nonclinical sample and a clinical sample of eating disorder patients. Psychologically controlling parenting involves the manipulation of the parent-child bond through the use of intrusive practices such as guilt-induction, shaming, and conditional regard [46]. In the context of controlling parenting practices, children's basic psychological needs are likely to be thwarted, as parents force their children to comply with their agenda (autonomy-thwarting), as children feel unable to meet parents' expectations (competence-thwarting), and as the use of psychological control creates distance and coldness in the parent-child relationship (relatedness-thwarting) [47]. Soenens et al. [45] showed that this need-thwarting parenting style was associated with more maladaptive perfectionism, which, in turn, predicted drive for thinness, body dissatisfaction and bulimic symptoms in both clinical and nonclinical samples. Further, the clinical sample reported experiencing more paternal psychological control relative to the non-clinical sample. In another study, Soenens and colleagues [48] demonstrated that parental psychological control is not only associated with more rigid and self-critical functioning concurrently, but also predicts an increase in such functioning over time, which, in turn, predicts a rise in depressive symptomatology. Together, this body of work suggests that, as the result of being exposed to a critical, pressuring, and cold parenting climate, individuals may become increasingly self-critical, such that they rigidly stick to high standards for thinness and physical attractiveness. On the other hand, a need supportive parenting style is associated with more need satisfying experiences and fewer body image concerns and disturbed eating behaviors.

Some studies more directly examined the link between need satisfaction and need thwarting and disordered eating (path B in Figure 1). In a first study, it was found that adolescent athletes who experienced more psychological need thwarting during sport activities reported more eating disorder symptoms [49]. Further, people who reported their psychological needs were not satisfied experienced a stronger urge to eat and more binge eating behaviors [50]. These relations have not only been established at the interpersonal level, but also at the within-person, day-to-day, level. In a diary study, Verstuyf and colleagues [Verstuyf J, Vansteenkiste M, Soenens B, Boone L, Mouratidis A: Daily ups-and-downs in healthy eating and binge eating symptoms: the role of psychological needs, emotional eating and general self-control strength, submitted] found that daily fluctuations in psychological need thwarting are associated positively with daily fluctuations in binge eating symptoms, whereas daily fluctuations in psychological need satisfaction were associated positively with daily fluctuations in healthy eating behaviors. Finally, Thøgerson-Ntoumani and colleagues [44] found evidence for a path model in which psychological need satisfaction was associated with less body dissatisfaction and drive for thinness, which, in turn was predictive of unhealthy weight behaviors, such as skipping meals and purging (see path C in Figure 1).

A third group of studies examined the association between one's general self-determined motivation and the endorsement of the thin-ideal as well as eating regulation outcomes (paths E and F in Figure 1). Pelletier and colleagues [43,51] found that young women's general disposition to act in a self-determined way protects them against the adverse effects of sociocultural pressure to be thin and is negatively predictive of their tendency to endorse the thin-ideal. As a consequence, those who function in more self-determined ways were found to be less likely to engage in disordered eating behaviors (e.g., bulimic symptoms) and more likely to engage in healthy eating behaviors (e.g., amount of vegetables eaten). In a similar study, Kopp and Zimmer-Gembeck [52] reported negative associations between general self-determination and perceived sociocultural pressures to be thin and adoption of the thin-ideal. In line with these studies, Mask and Blanchard [53] found that women who are in general more self-determined, did not report body image concerns when exposed to a video portraying the female body as an object, whereas women low in general self-determined motivation reported more negative self-appraisals, body shame, and internally pressuring motives for eating when faced with such a body-objectifying situation [see also Mask L, Blanchard CM: The Differential Role of Autonomous and Controlled Motivation Against Body-Object and Body-Process Media on Women's Body Image Concerns and Eating Behaviors, submitted].

#### Need Substitutes and Compensatory Behaviors: Clinical Implications and Future Research Directions

Although limited, studies within the context of eating regulation suggest that need thwarting is associated with (a) a stronger focus on appearance and body image and (b) more compensatory behaviors, such as uncontrolled eating and rigid functioning. In contrast, general self-determined motivation buffers against sociocultural pressures to be thin, personal endorsement of the thin-ideal and disordered eating behaviors.

Many issues remain to be addressed in future research, including the necessity to examine the role of need thwarting in the development of need substitutes, compensatory behaviors, and eating regulation problems over time. These variables might be reciprocally related to each other such that the pursuit of need substitutes and the engagement in compensatory behavioral patterns predict need thwarting over time which, in turn, is predictive of an increasing focus on need substitutes and compensatory behavioral patterns. Another question that remains unaddressed is why some people indulge in rather uncontrolled eating behaviors when faced with need thwarting experiences, whereas others develop a more rigid and controlled coping strategy to such experiences. It would be interesting to investigate whether individuals' general tendencies toward self-determined functioning (e.g., causality orientations) [54], would help to clarify the circumstances under which these different behavioral responses are likely to emerge.

Although more research is needed, previous findings suggest that individuals' experienced degree of need satisfaction and need thwarting is involved in their eating behaviors. This implies that health-care providers can guide eating disordered patients to a healthier eating style by supporting their psychological needs. For instance, health care providers can help patients detect need-satisfying and need-thwarting sources in their life. For some, it might be useful to discuss need-thwarting experiences in their past, as a means to alleviate disruptions caused by these experiences. Also, learning effective coping tools to handle need thwarting experiences could prevent patients from engaging in maladaptive coping mechanisms, such as compensatory behaviors and pursuing need substitutes. Further, treatment contexts that are need-supportive in both one-on-one counseling experiences as well as at the level of the treatment facility can enhance clinical outcomes given the beneficial psychological milieu already demonstrated to result from need-supportive experiences in other contexts [44,55]. Some empirical evidence already exists to support the importance of need satisfaction in clinical treatment of disordered eating. Creating a more need supportive context in a residential setting for patients with an eating disorder resulted in greater treatment engagement and less treatment dropout [56]. Motivational interviewing can offer more insights into how to work with clients to become more self-determined in treatment [57]. The emphasis on personal choice, empathy, and competence can contribute to a sense of need satisfaction, which, in turn, relates to a healthier and less disordered eating style.

### The Role of Psychological Needs in the Optimal Regulation of Eating Behaviors

#### Optimal Regulation of Eating Behaviors: A Self-Determination Theory Perspective

In addition to playing a relatively *distal *role in the etiology of eating disordered behaviors and attitudes, processes of need satisfaction and need thwarting may also be more *proximally *involved in people's ongoing regulation of food intake and weight. The way eating behaviors are regulated and the experiences during the regulatory behaviors will depend on (a) individuals' motivational regulation of eating behavior and (b) the goals underlying eating regulation (see paths A-D in Figure 2). Notably, the motivational basis for one's ongoing eating regulation may be very different for individuals who display a general self-determined motivation style compared to individuals who score high on need substitutes and compensatory behaviors (see path G and H in Figure 1). Thus, processes discussed in the previous section also are associated with one's motives and goals for the ongoing regulation of eating behaviors.

##### Regulation of Eating Behavior

In its focus on motivational quality, SDT has conceptualized the types of motives underlying a variety of behaviors and endeavors, including one's eating regulation [15]. Early research on motivation focused on the distinction between behaviors that are intrinsically versus extrinsically motivated [58]. Intrinsic motivation refers to undertaking an activity for its inherent interest and enjoyment, whereas extrinsic motivation refers to engaging in an activity to achieve an outcome separable from the activity. The concept of intrinsically motivated behaviors is embedded within the view that people are inherently active organisms with a natural tendency toward growth and development, with intrinsic motivation being a manifestation of this growth-tendency. However, not all behaviors are inherently interesting or pleasurable. This might be the case particularly in the context of eating regulation, where perhaps few individuals restrict their food intake or adopt a different eating pattern because they find it inherently enjoyable to do so. Changing one's eating behaviors often involves some degree of physical and/or psychological discomfort and, although some individuals might develop an interest in their daily eating pattern or might perceive changing their eating behavior as a positive challenge [17,59], many individuals might not be intrinsically motivated to regulate many or most of their eating behaviors. Indeed, attempts to change eating patterns that are directed toward some separable outcome - whether that is to improve health, lose weight, or attain a more desirable physique - are by definition extrinsically motivated. However, there exists considerable variability in the extent to which the reasons underlying one's extrinsically motivated behavioral change are self-endorsed, that is, internalized within people's broader goals and values. Therefore, SDT has distinguished different types of extrinsic motivation that fall along a continuum of increasing autonomy and volition [16]. Behaviors that are more controlled are carried out with a sense of pressure and coercion whereas those that are more autonomously regulated are characterized by a sense of personal endorsement and internal consistency [15,60].

The most controlled form is external regulation, which refers to carrying out an activity to conform to other people's demands. The behavior is oriented toward attaining positive outcomes, like others' approval or a promised reward, or to avoid negative outcomes, like criticism or threatening punishments. These types of outcomes can be explicit and clear, but they can also be implicit or subtle, and thus hard to identify, even for the person/group in question. The second controlled form of regulation is introjected regulation whereby a behavior is regulated based on internal pressure such as feelings of guilt, shame, or contingent self-worth [15]. For both external and introjected regulation, the behaviors are accompanied by feelings of pressure and obligation.

Identified and integrated regulation represent two relatively more autonomous forms of extrinsic motivation [15,16]. Identified regulation refers to carrying out a behavior because one understands and values the importance of this behavior. Integrated regulation involves not only valuing the behavior but also bringing it in harmony with one's other goals and values. In both cases, one has the feeling of 'wanting' instead of 'having' to change one's eating behaviors.

This motivational continuum has been used to predict a range of outcomes, including performance, persistence, and psychological well-being, across several domains, including work [61], education [62], sports and exercise [63], psychotherapy [64], and health care [60]. More autonomously regulated behaviors have been found to engender a sense of vitality and energy and were found to relate to more need satisfying experiences within a given context. For instance, autonomous motives for work were associated with more need satisfying experiences at work, which, in turn, was associated with more vigor, job satisfaction, and better performance. In contrast, more controlled motives were associated with less need satisfying experiences which, in turn, predicted exhaustion and lower performance [19]. Similarly, in the context of eating regulation, more autonomous motives can elicit more need satisfying experiences during the process of eating regulation which, in turn, is associated with more energy and sustained healthy eating. In contrast, a controlled eating regulation would evoke more need thwarting experiences during the process of eating regulation and therefore deplete one's energy and resources for successful eating regulation (see path C and F in Figure 2).

##### Goals Underlying Eating Regulation

Consistent with the differentiation between intrinsic and extrinsic aspirations at the global level described above, people can pursue intrinsic or extrinsic goals when regulating their eating patterns. For example, someone can attempt to change his eating habits mainly to obtain a desirable physique (extrinsic goal) or mainly for the purposes of becoming healthier and more fit (intrinsic goal). Although in practice both goals might be present to some extent, the relative importance attached to these two types of goals yields a different relationship to eating behaviors. In the context of leisure-time physical activity, the more importance was attached to health relative to appearance, the more one experienced leisure-time physical activity as need satisfying which, in turn, was related to higher physical self-worth, higher well-being, and less exercise anxiety [65]. Similarly, an appearance-focused eating regulation is said to evoke more need thwarting experiences which, in turn, relates to more unhealthy and disturbed eating patterns (path D and F in Figure 2).

#### Optimal Regulation of Eating behaviors: An Overview of Empirical Evidence

To date, a handful of studies have examined the role of general motivational functioning in predicting motivation for eating regulation (paths G and H in Figure 1). Pelletier and Dion [43] found that general self-determination was positively associated with more autonomous regulation for eating behaviors and negatively associated with more controlled eating regulation. Also, body dissatisfaction was associated with more controlled forms of eating regulation, whereas it had no association with more autonomous forms of eating regulation [52,66,67]. Finally, when faced with events that trigger body dissatisfaction, more self-determined women do not appear to develop introjected motives for eating regulation [Mask L, Blanchard CM: The Differential Role of Autonomous and Controlled Motivation Against Body-Object and Body-Process Media on Women's Body Image Concerns and Eating Behaviors, submitted]. The motives and goals for eating regulation, in turn, influence how one regulates eating behaviors and the probability of succeeding or failing in one's dietary attempts (see Figure 2).

##### Motivational Regulation of Eating Behavior

Several studies have provided evidence for associations between the motives for eating behaviors and healthy or disordered eating behaviors. Pelletier and colleagues [68] found that autonomous eating regulation was associated with more healthy eating (e.g., eating more vegetables and fruits) and fewer bulimic symptoms. In contrast, controlled eating regulation was associated with less healthy eating and more bulimic symptoms. Interestingly, autonomous eating regulation was associated with being concerned with *what *one eats (i.e., quality of one's food), whereas controlled eating regulation was associated with being concerned with *how much *one eats (i.e., quantity of food) (see also [43]). Further, autonomous eating regulation significantly predicted a reduction in percentage of calories from total and saturated fats over a 26-week period [68]. In line with these findings, a study with participants in a commercial weight loss program found that an autonomous eating regulation related to eating more fruits and vegetables, whereas controlled eating regulation had no associations with eating behaviors [69]. Other research has examined the mechanisms through which autonomous and controlled eating regulations affect eating behaviors (see path A in Figure 2). These studies are interesting as they might provide more insight into why a preponderance of autonomous, relative to controlled, regulations is experienced as more need satisfying (see path E in Figure 2). For instance, Otis and Pelletier [70] found that autonomous eating regulation was associated positively with approach food planning (i.e., planning to eat more healthy foods), whereas controlled eating regulation was associated positively with avoidance food planning (i.e., avoiding too many calories, certain kinds of foods). Both approach and avoidance food planning were shown to be significant mediators of the associations between autonomous or controlled regulation and healthy eating behaviors, with approach food planning being positively predictive and avoidance food planning being negatively predictive of healthy eating behaviors (see path F in Figure 2). Further, it has been found that highly controlled, relative to highly autonomous, dieters display more extreme and rigid dieting behaviors across a 5-month period [71]. In turn, flexible, relative to rigid, restrained eating has been shown to predict successful weight control, especially in the long-term [72]. Finally, Hagger, Chatzisarantis and Harris [73] found that autonomous motivation for dieting predicts a more positive attitude toward dieting and more perceived behavioral control over eating behaviors. Collectively then, this set of studies suggests that having an autonomous, relative to a controlled regulation of one's eating behavior is associated with a different approach towards one's eating behavior. This, in turn, may be associated with different experiences of need satisfaction or thwarting (see path E in Figure 2). For instance, an avoidance-orientation in goal pursuit has been found to predict less competence and autonomy [74]. These ideas await further empirical testing in the context of eating regulation.

A few studies have investigated the relative effects of autonomous and controlled motivation for changing one's eating behavior, in the context of clinical weight loss treatment. For instance, Williams and colleagues [75] found that being autonomously motivated to enter a weight management program was associated with greater program attendance and greater weight loss at the end of the intervention, in a sample of obese adults. Also, in a sample of overweight and obese women, autonomous treatment motivation was associated positively with improvements in eating self-efficacy and cognitive restraint and was associated negatively with disinhibition, emotional, and external eating [76]. Within the same trial, it was also observed that controlled regulation to enter obesity treatment was associated with poorer body image and lower psychological well-being [67] and that 1-year changes in weight loss treatment motivation predicted changes in psychological well-being in overweight women in the expected direction [77]. Also, intervention studies have found that experimentally increasing autonomous motivation for changing eating behaviors during treatment results in more weight loss compared to individuals in a control group for those who had a controlled motivation for dieting at baseline [78]. This set of studies suggests that considering the motivational dynamics underlying eating behavior change and promoting autonomous eating regulation are important for weight loss treatment.

##### Eating Regulation Goals

In addition to the motives for eating regulation, SDT maintains that it is critical to examine the goals underlying eating regulation, as different goals can elucidate different motivational dynamics. A first study demonstrated that dieting out of concern for one's appearance was associated with more drastic dieting strategies and with losing control over eating [79] (see path C in Figure 2). Another study demonstrated that both health-focused and appearance-focused weight loss goals in a group of overweight participants are associated with the number of diets, but that only appearance-focused weight loss goals were associated with the frequency of binge-eating episodes [80]. Two other studies demonstrated that the pursuit of a slender and physically attractive body through dieting was associated with more diet-specific need thwarting and unhealthy weight behaviors, while the pursuit of a healthy and fit lifestyle was associated with less diet-specific need thwarting and unhealthy weight behaviors [44,66] (see path D and F in Figure 2).

#### Eating Regulation Motives and Goals: Clinical Implications and Future Research Directions

Together, previous studies suggest that it is important to consider the motivational basis for eating regulation as this is related to the success or failure of eating regulation with regard to weight loss and problematic eating behaviors. In line with SDT's basic tenets, autonomous versus controlled eating regulation, and the pursuit of health versus physical attractiveness, have been associated with more adaptive outcomes such as a more flexible approach to eating regulation, less diet-specific need thwarting, and more healthful and less disordered eating.

Future research is needed to more clearly elucidate the processes through which these motivational variables influence eating regulation. For instance, the association between the motives for eating regulation and experiences of need satisfaction or thwarting has not been addressed directly in previous research. Also, reciprocal relations between a rigid and avoidance-oriented approach to eating and need thwarting experiences during the regulatory process, still await empirical testing. Further, given the paucity of studies on intrinsic and extrinsic goals in the context of eating regulation, future research can investigate whether a focus on appearance versus health is associated with an increase in unhealthy or problematic eating behaviors over time and can shed light on the processes that can account for these differential associations. Finally, more research is needed to investigate how, across time, the motives and goals for eating regulation, diet-specific need thwarting, and eating behaviors affect each other in a reciprocal and mutually reinforcing fashion.

At the clinical level, the current research base suggests that health care providers could help patients evolve to a more healthy eating style by stimulating an optimal motivational quality for eating regulation. For instance, physicians and nutritionists could start from the patients' perspective rather than imposing a dietary plan. Patients can be informed about health risks associated with overweight, while health care providers simultaneously keep an open view on the patients' perspective and their reasons to change and not to change. Further, research has shown that a need-supportive context enhances more autonomous forms of behavioral regulation [64,81]. Therefore, creating a need-supportive context at the organizational and therapeutic level, can also improve one's ongoing eating regulation. Motivational Interviewing [82] provides a practical set of intervention guidelines, skills, and strategies which are well-developed, field-tested, and are largely consistent with SDT premises on motivation and lasting behavior change, including changes in diet [57].

### SDT in Relation to Current Perspectives on Body Image Concerns and Eating Regulation

Although little research to date has examined motivational dynamics in eating regulation, many extant and intensively examined models of eating regulation have conceptual overlap with some of the basic tenets of SDT. In this section, we discuss SDT in relation to some of the prevailing perspectives on body image concerns and eating regulation, thereby focusing on how SDT-based constructs and processes may add to an understanding of the motivational dynamics in the context of eating and weight regulation. It is not our aim to exhaustively review and discuss the wide variety of models developed in the context of eating regulation (see [14] for an overview), but rather to selectively discuss those models where the motivational perspective of SDT can contribute to a more thorough understanding of how eating behavior is self-regulated. Furthermore, because the models are mainly discussed in relation to SDT, they are only briefly summarized.

#### Thin-Ideal Internalization Model

Various scholars (e.g., [2]) have emphasized the critical role of sociocultural influences in the adoption of the thin-ideal, which represents a risk factor for the development of body dissatisfaction and disordered eating regulation. In much of Western society and other parts of the developed world, people (particularly women) are bombarded with images of thin and attractive models through advertisements and mass media [24]. When exposed to such images, people feel pressured to adopt the thin-ideal as a personal goal. Cross-sectional, longitudinal, and experimental studies have provided evidence for this effect, demonstrating that individuals who experience sociocultural pressure to be thin are more likely to aspire to the thin-ideal and to experience body image concerns [6,83].

Although SDT and the Thin-Ideal Internalization model use different terminology, there is considerable overlap between the concept of extrinsic goals within SDT, and more specifically physical appearance goals, and the concept of adoption of the thin-ideal. Pursuing the thin-ideal can be considered as a more extreme form of pursuing physical attractiveness, in which the norm for physical appearance is socially prescribed and more difficult to attain. Both SDT and the Thin-Ideal Internalization model acknowledge the flimsy promise that achieving attractiveness will result in increased well-being, control, and freedom [14,84,85]. SDT explains the fleetingness of this promise with its conceptualization of extrinsic goals, which may result in derivative satisfaction when the ideal is achieved but creates a very unstable form of well-being as it is unlikely that achieving the thin-ideal contributes to genuine need satisfaction.

Different from the thin-ideal internalization model, SDT also provides an alternative to this less-fulfilling extrinsic goal in the form of intrinsic goals and aspirations. From the perspective of SDT, people may pursue weight management and eating regulation in less functional ways - by striving for unattainable ideals propagated by images in popular media - or in more adaptive ways - by pursuing health and physical fitness. Because SDT provides this positive alternative in the form of intrinsic goals, it also incorporates more positive indicators of well-being (e.g., positive affect, vitality) [22]. This is in contrast to traditional perspectives such as the thin-ideal internalization model that typically focuses on body dissatisfaction and disordered forms of eating as outcomes (e.g., [6]).

Another similarity between the thin-ideal internalization model and SDT is that both emphasize the critical role of the social environment in the adoption of the thin-ideal. When individuals are repeatedly exposed to images and messages that the pursuit of the 'perfect body' yields happiness, they may model their own behavior and aspirations accordingly. One intriguing question is whether some individuals are more susceptible to the experience of sociocultural pressure and to the subsequent pursuit of the thin-ideal compared to others. SDT's perspective on need satisfaction and need thwarting may offer some insights in this regard. When people's basic needs have been chronically thwarted, they might feel more insecure which, in turn, may lead them to pursue need substitutes in an attempt to compensate for thwarted needs. One possibility is that when individuals experience need-thwarting, they may seek out distractions in the form of television, fashion magazines, and other forms of media that expose them to advertisements promoting the thin-ideal [75]. This increased exposure to sociocultural norms for thinness may make them more susceptible to these messages. An alternative possibility is that both need-thwarted and need-satisfied individuals are equally exposed to such media, but that need-thwarted individuals interpret the message as more pressuring and controlling. A third possibility is that need-thwarted and need-satisfied individuals interpret the same ads as equally pressuring, but that need-satisfied individuals cope differently with these pressures. Need-satisfied individuals might more easily question the message spread by the mass media and may reflect on whether the pursuit of thinness fits with their own preferences and goals. In contrast, need-thwarted individuals might more readily accept the "truth" of these messages and, as a result, endorse the thin-ideal more strongly. Some research to date has found that women who are in general more self-determined experience less sociocultural pressures, adopt the thin-ideal less strongly and even react differently to equally pressuring images [51,53]. Future research is needed to better clarify the role of need satisfaction in susceptibility to and endorsement of the thin-ideal.

Importantly, the adoption of cultural messages such as the thin-ideal might also interfere with the potential to experience subsequent need satisfaction [66]. The pursuit of physical attractiveness and the thin-ideal in particular promote an outward orientation, such that individuals hinge their self-worth and value upon achieving this ideal. This kind of goal pursuit creates intrapersonal pressure (reduced autonomy) and may lead these individuals to engage in stressful and potentially socially-alienating social comparisons (lack of relatedness). Failure to achieve the unattainable goals set up by social norms and pursuit of the thin-ideal often results in feelings of inferiority stemming from an inability to reach one's goals (lack of competence). More research is needed to identify how need satisfaction and thwarting function as both antecedents to and consequences of adoption of the thin-ideal. Future research incorporating elements of both SDT and the thin-ideal internalization model is important for further clarifying the potential overlap of and distinction between these two perspectives as they relate to body satisfaction and eating regulation.

#### Self-Objectification Theory

Another model that elaborates on the role of sociocultural influences in body image concerns and eating disorders is *Self-Objectification Theory *[11]. Within this theory, girls and women are said to measure their self-worth by evaluating their physical appearance against the sexually objectifying and often unrealistic standards of beauty that prevail in Western society. Western culture is said to socialize girls and women in such a way that they take a third-person or observer perspective toward their own body, which makes them preoccupied with their appearance and leads them to objectify their own body. Consistent with the theory, several studies have shown that trait self-objectification is associated with depression, body shame, and bulimic and restrictive eating disorders [11,86]. In addition to these more stable interpersonal differences in self-objectification, certain situations (e.g. trying on a swimsuit) can trigger self-objectification. Such primed self-objectification yields an array of negative consequences, including body shame, restrained eating [87], and impaired performance [88].

Although self-objectification theory is embedded within a feminist perspective and SDT stems from motivational psychology, there are some interesting conceptual similarities that are worth noting. For instance, both theories state that a preoccupation with physical appearance will have negative effects for people's general (e.g., depression) and domain-specific (e.g., body shame, unhealthy weight behaviors) functioning. Notably, in both frameworks, the relative importance of physical appearance compared to other goals is emphasized. The measurement of trait self-objectification [88] requires individuals to rank order a set of 12 body attributes, half of which reflect a preoccupation with physical appearance and half of which reflect a focus on physical competence, such as health, energy level, and physical fitness. Similarly, studies within the context of exercise of eating regulation that investigated one's goal orientation, often compared the relative importance attached to health versus appearance [65,66].

Additionally, both frameworks emphasize the adverse role of objectification. Within SDT, the concept of objectification has been proposed as a mediating mechanism between one's goal orientation and need satisfaction [23,25]. Specifically, the adoption of an objectifying stance toward others is characterized as dehumanizing [89] because it reduces others to objects. The target of this objectification process might be different depending on the specific nature of the extrinsic goal, with others being objectified if someone strongly values materialism, power, or fame and with one's own body being objectified if someone strongly values physical appearance and slenderness [25]. At a broader level, the adoption of an objectifying stance reflects a conditional approach to others' or one's own body. An example from another domain may serve to illustrate this point. People who strongly value money and power may appreciate others only to the extent that they can help them in achieving their extrinsic ideals. Similarly, people who strongly pursue physical attractiveness may appreciate and value their body only when they meet the expectations of being attractive, but feel ashamed of their body and disappointed in themselves when they fail to meet this objective.

(Self-) objectification also precludes a full investment in the regulatory activity at hand. Consistent with the experimental work within Self-Objectification Theory [88], Plant and Ryan [90] demonstrated that dispositional and experimentally induced public self-consciousness, which reflects individuals' tendency to be aware of themselves as objects of others' observation, yielded deleterious effects on individuals' enjoyment of the activity. More recently, the framing of an activity to achieve an extrinsic goal, relative to an intrinsic goal, has been found to disrupt conceptual learning, because extrinsic goals put pressure on individuals and forego a task-involved approach of the learning activity [62]. A similar explanation has been provided within Self-Objectification Theory: the negative effects associated with the induction of state self-objectification are said to result from the constant monitoring of one's body which is said to interfere with full absorption in other activities (e.g., work [11]). From the SDT perspective, continual distraction from the activity will likely undermine the satisfaction of one's basic psychological needs for autonomy, competence, and relatedness, such that the energetic resources needed for the ongoing eating regulation are more easily eroded.

Despite these similarities, there are also some differences between the SDT framework and self-objectification theory. First, whereas self-objectification theory explicitly focuses on self-objectification in the context of appearance, SDT considers pursuing physical attractiveness as one type of extrinsic goal that has adverse effects on people's functioning and well-being. Further, SDT explains the harmful effects of objectification in terms of its association with basic psychological need satisfaction. Nevertheless, and in light of the correspondence between self-objectification theory and SDT, it would be interesting for future research to directly examine whether self-objectification could play an explanatory role in the relationship between goals and diet-specific need thwarting and maladaptive eating behaviours.

#### Dietary Restraint Theory

Advertisements and the media strongly emphasize the idea that the 'thin-ideal' can be achieved by dieting [14]. Given the positive meaning attached to the thin-ideal, it is not surprising that the dieting industry has boomed [14] and that the majority of adolescent girls [91] and adult women [92] indicate they have dieted or are currently dieting to lose weight. Unfortunately, it is uncertain whether dieting has the expected positive effects on individuals' weight and body size. This is because many people who start dieting fail to control their food intake adequately [93]. For instance, several diet programs have been unsuccessful in promoting long-term weight loss [94,95]. According to Dietary Restraint Theory [12], dieting can even be a causal factor contributing to overeating and bulimic symptoms. Much research attention has been devoted to this issue but results are mixed and it remains unclear whether dietary restraint should be recommended or discouraged to improve body image and regulation of eating behaviors [96]. SDT may provide some useful insight into when and why dieting is more likely to fail.

According to the Dietary Restraint Theory [12], dietary restraint can have adverse effects on food intake and result in overeating. Heightened attention to food intake can create a cognitive boundary, which replaces a more intuitive regulation of food intake. This overly-cognitive focus reduces people's sensitivity toward physiological signs of satiety and hunger and instead creates a preoccupation with psychological, cultural, or social signs to eat [41]. In line with this claim, experimental research [97] showed that individuals high in dietary restraint were more likely to indulge in overeating after having violated their cognitive rules about food intake (e.g., after eating a small amount of high caloric food). The process whereby dieters lose control over their food intake is known as the "disinhibition effect" [97]. The dietary restraint hypothesis has been incorporated within the Dual Pathway Theory [6] as one of the pathways toward the development of bulimic symptoms, particularly bingeing.

Although the Dietary Restraint Theory does not explicitly focus on motivational dynamics underlying dieting efforts, some processes that have been proposed to understand the disinihibition effect can be linked to one's motivation for eating regulation in our view. For instance, some dieters display a shift in cognitions, vacillating from restrictive restraint to giving in to their urge to eat or even actively rebelling against self-imposed dieting rules [41,98]. Research within SDT has shown that a breakdown in one's self-regulatory activities and rebellious actions against (self)-imposed rules are more likely to result from a controlled, rather than autonomous, regulation [21]. Second, we suggest that the all-or-nothing approach to dieting ('once I break a diet rule, the entire process becomes worthless') described in dietary restraint theory as the abstinence-violation effect [99] can be linked to a controlled regulation of one's behaviors hinging one's self-worth on a regulatory activity or goal (i.e. introjected regulation). Third, the rebound-effect [100], which is the increase in thoughts about eating [101] and eventually actual eating [102] after having suppressed thoughts about 'forbidden' foods, is most likely to occur in dieters with a controlled regulation for dieting. That is, dieters with a controlled motivation for eating are more likely to use avoidance strategies (e.g., avoiding foods that are high in fat) to change their eating behaviors [70]. Dieters with a more autonomous eating regulation will more often use approach goals such as eating more healthy foods.

In sum, although Dietary Restraint Theory maintains that dietary restraint can result in a disinhibited eating style, research has shown this it is not necessarily the case. Although motivational dynamics are not explicitly discussed in this model, the processes that are found in dietary break-down are more closely connected to a controlled pattern of eating regulation. Future research could more explicitly investigate motivational dynamics underlying dietary restraint and investigate whether the differentiation between several types of motivation (goals and regulatory styles) can promote more insight into when and why dietary restraint is likely to fail.

#### Self-Control Theory

Self-Control Theory [13] hypothesizes that eating regulation will fail over time. Based on their self-regulation or self-control model, Baumeister and Heatherton [13] argued that people's self-control capacity is a limited resource or strength that gets depleted over time (i.e. ego-depletion). Self-control is defined as "the use of cognitive and attentional resources to override, inhibit, or alter impulses in the service of attaining personal goals or satisfying motives" [p. 94:214]. According to self-control theory, self-control is a limited resource that can be used up, although there is individual variation in people's resources available for self-control. From the self-control perspective, eating regulation can be considered as one form of behavioral control [103]. Behavior control is seen as psychologically demanding and, hence, will use up people's self-regulation resources. This implies that dieters would be successful in regulating their eating pattern as long as they have sufficient resources available for self-control. However, resources for eating regulation would become depleted when people need to regulate for longer periods of time or when situational demands challenge their self-regulation efforts. Consistent with this reasoning, research has shown that dieters ate more high caloric food when they had already consumed their self-regulatory resources on a previous (even unrelated) task [103,104].

SDT concurs with self-control theory that eating regulation can involve effort and be both psychologically and physically draining. Although for some people changing eating behaviors is perceived as an intrinsically motivated challenge, for most it is probably an extrinsically motivated behavior in the service of attaining a separable goal (e.g., losing weight, becoming more attractive, increasing fitness, or feeling better). An important difference between both frameworks is that, according to SDT, the ego-depleting character of eating regulation will depend on the motivational basis for eating regulation. Because of the differential relationship with the three needs, a controlled and appearance-focused eating regulation is more likely to be ego-depleting [105,106]. In contrast, autonomous and health-focused eating regulation is less likely to be resource-depleting, and the fulfillment of psychological needs is likely to be resource-restorative. Indeed, research has demonstrated a positive link between autonomous self-regulation and subjective vitality (i.e. experiencing psychological energy). For instance, severely obese patients who entered treatment with a more autonomous motivation for behavior change reported higher levels of subjective vitality at the 2-year follow-up ([107] study 5). Also, persistence in ego-depleting activities, such as elite swimming, is higher amongst autonomously motivated individuals [108]. Further, experimental studies have shown that individuals being placed in a controlling, relative to those being placed in an autonomy-supportive environment, experience greater ego-depletion after exerting initial self-control [106]. Similarly, Moller et al. [105] found that making choices yielded an ego-depleting effect when the individual felt pressured to choose a certain option, but found that the ego-depleting effect was absent in an autonomous choice condition in which participants freely chose their desired option. Moreover, these experiments indicated the effect of an autonomous versus controlled regulation on subsequent self-control in unrelated tasks was mediated by feelings of vitality ([106] Study3; [105] Study 3).

Together, these studies demonstrate that the ego-depleting effects of self-control depend on the underlying motives for exerting self-control. Less research has been conducted regarding the role of underlying goals in self-regulation. One study found that appearance-focused, relative to health-focused, eating regulation was associated with more diet-specific need thwarting, which in turn predicted more bulimic symptoms [66]. Also, appearance-focused exercising predicted more exercise-specific need thwarting and, in turn, was related to less perseverance of the exercise behaviors [65]. More research is needed to investigate whether the ego-depleting effects of eating regulation is dependent upon the motivational basis for eating regulation and, to investigate whether diet-specific need thwarting can explain why eating regulation is energy-draining. Further, although research demonstrated the differential effects of underlying motives for regulation on ego-depletion, future research needs to examine more directly the impact of goals underlying self-regulation on ego-depletion.

## Conclusion

Eating regulation encompasses a wide variety of behaviors that have been intensively studied over the past 30 years. Although specific processes are involved in different manifestations of eating regulation (e.g., weight management, purging, restraint), we argue that motivational dynamics represent a common factor underlying the range of eating behaviors. Specifically, SDT may be of added value to the eating regulation literature for two reasons. First, the concept of basic psychological needs, as conceived within SDT, can help to bridge different parts of the literature on eating regulation. This is because the satisfaction and thwarting of one's basic psychological needs for autonomy, competence, and relatedness represent key mechanisms to understand how disordered eating develops and how people manage or fail to optimally regulate their ongoing eating patterns. While many theories and models in the eating regulation literature have addressed either disordered eating or ongoing eating regulation, the concept of psychological needs represents a promising process to simultaneously address both issues.

Second, what is critical from the SDT perspective is to move beyond considering individuals' level or degree of eating regulation and instead adopt a more differentiated approach. This is achieved by distinguishing different types of motives (i.e., autonomous and controlled) and different goals (i.e., intrinsic and extrinsic) for eating regulation which have been found to yield distinct eating outcomes, in part because they allow for varying degrees of need satisfaction. We hope that this review encourages scholars in the field of eating regulation to devote greater attention to the motivational dynamics in eating regulation and to examine the overlapping and unique aspects of SDT in relation to existing frameworks in this field.

## Competing interests

The authors declare that they have no competing interests.

## Authors' contributions

JV, MV and HP discussed the format and scope of the manuscript. JV and HP wrote the initial draft of the manuscript. MV and PT revised the manuscript critically and contributed to the writing of the manuscript. All authors read and approved the final manuscript.
